# Comparison between Intravenous Sodium Valproate and Subcutaneous Sumatriptan for Treatment of Acute Migraine Attacks; Double-Blind Randomized Clinical Trial

**Published:** 2014-03

**Authors:** Abolghasem Rahimdel, Ali Mellat, Ahmad Zeinali, Elahe Jafari, Parisa Ayatollahi

**Affiliations:** Department of Neurology, Yazd University of Medical Science, Yazd, Iran

**Keywords:** Migraine, Sodium valproate, Sumatriptan

## Abstract

**Background:** Sodium valproate (SV) has been approved for migraine prophylaxis and its intravenous form is used to treat acute migraine attacks. We compared the efficacy and safety of intravenous SV and subcutaneous Sumatriptan in managing acute migraine attacks. **

**Methods:** This double-blind randomized clinical trial divided 90 patients into two groups: one group received 400 mg of intravenous SV and the second group received 6 mg of subcutaneous Sumatriptan. Headache severity before treatment and half an hour, one hour, and two hours after treatment was measured based on the VNRS in the groups. Associated symptoms, i.e., photophobia, phonophobia, nausea, and vomiting, were assayed on admission and 2 hours after treatment. Side effects of the drugs were checked 2 hours after injection. Obtained data from the groups were compared.**

**Results:** In both groups, pain decrement at the mentioned time points was significant (P<0.001), but had no significant difference (P>0.05), indicating the similar effect of both drugs on pain improvement. In the SV group, photophobia, phonophobia, nausea, and vomiting were improved significantly, while in the Sumatriptan group, only photophobia and vomiting were decreased significantly, indicating the advantage of SV in improving the associated symptoms. Nausea, vomiting, facial paresthesia, and hypotension were more significantly frequent in the Sumatriptan group than in the SV group (P<0.05).

**Conclusion:** Intravenous SV (400 mg) was as effective as subcutaneous Sumatriptan in the treatment of acute migraine attacks, but with more improvement in associated symptoms and with fewer side effects.**

**Trial Registration Number:** IRCT201108025943N4

## Introduction

Migraine commonly presents as a unilateral (60%), pulsatile (85%) headache which is usually associated with nausea (90%), vomiting (30%), photophobia and phonophobia (80%), and fatigue.^1^ Age shows a bimodal distribution in men and women, peaking in the late teens and 20s and around 50 years of age.^2^ The male-to-female ratio is 1/1 before puberty and 1/3 after puberty.^3^ The comorbidities of migraine are psychiatric (depression), neurological (narcolepsy), cardiovascular (patent foramen ovale), and others (fibromyalgia). Also, migraine has been known as a risk factor for other diseases such as panic attack, asthma, myocardial infarction, and depression.^4^

Migraine has two forms: classic (with aura) and common (without aura). The pathogenesis of aura and migraine headache is intracranial vasoconstriction and extracranial vasodilatation, respectively.^1^ Recent studies have revealed that focal cerebral ischemia occurs during migraine attacks. Vascular changes in migraine are secondary to primary dysfunction in the brain stem neurons.^1^ The predisposing factors for migraine attacks include  neck muscle pain, alcohol or coffee consumption, smoking, chronic stress, physical inactivity, hormonal changes, being female, low socioeconomic status and educational level, depression, sleep disturbance, obesity, diet (tyramine, monosodium glutamate, chocolate, nuts, and dried fruits), sudden changes in weather, hot and humid climates, bright light, and consumption of painkillers, oral contraceptive pills, or drugs such as dipyridamole and Trinitroglycerin.^1^^,^^5^^-^^9^

The treatment depends on whether migraine is acute or chronic. Patients with headaches lasting for more than 4 days per month may need prophylactic drugs.^10^ Nowadays, the most prevalent prophylactic drugs are Propranolol, sodium valproate, Topiramate, Amitriptyline, Verapamil, Gabapentin, Cyproheptadine, and Pizotifen.^11^^-^^14^ Acupuncture, relaxation therapy, biofeedback, and cognitive behavioral therapy also may have some benefits.^15^ Anti-migraine attack treatments should be affordable and cost-effective with few side effects and bring about speedy recovery and return to daily function and reduction in relapse.^16^^,^^17^

Oral sodium valproate was previously believed to be useful for prophylaxis in chronic migraine.^18^ In recent years, however, intravenous sodium valproate has been studied as anti-migraine attack treatment with good response.^19^^-^^23^ Because of the side of effects and contraindications of Sumatriptan, we sought  to compare the efficacy of sodium valproate and Sumatriptan in the treatment of acute migraine attacks.**

## Patients and Methods

**Figure 1:**
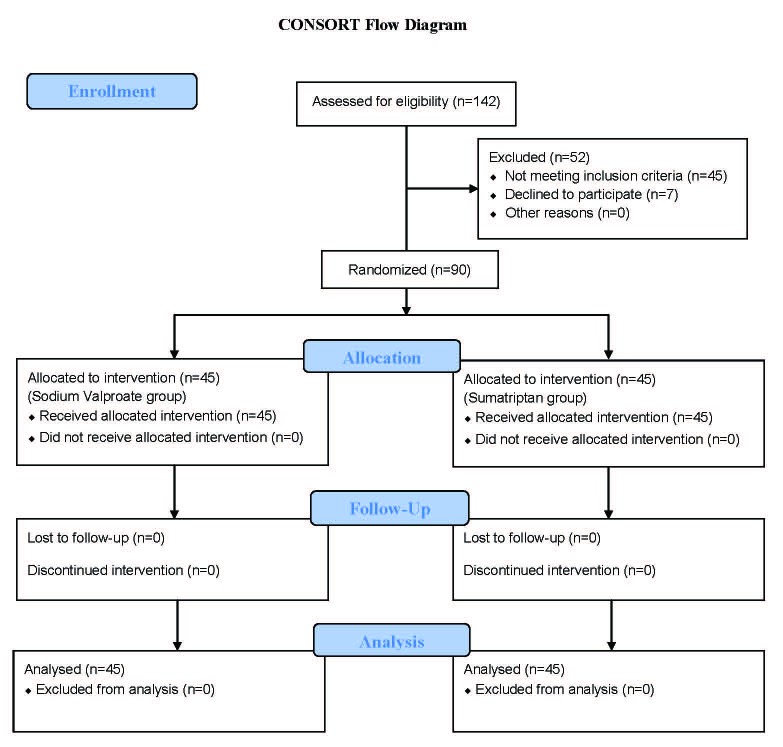
The patient’s consort flow chart is illustrated above.

This double-blind randomized clinical trial (IRCT Code: IRCT201108025943N4) was performed from December 2011 to May 2012 on 90 patients who referred to Shahid Sadoughi Hospital, Yazd, *Iran*. A simple randomized sampling procedure was performed on the basis of the study criteria using the “Random Allocation Software” program (figure 1). The patients, nurses administering the drugs, and those registering the signs and symptoms of the patients were blind to the medicine used in each group. The inclusion criteria were comprised of patients aged between 15 and 50 years with common migraine attacks and normal physical examinations. The exclusion criteria consisted of hepatic disease, familial medical history of hepatic failure, special forms of migraine such as hemiplegic, basilar, ophthalmic, and retinal, uncontrolled hypertension, coronary artery disease, unstable angina, peripheral vascular disease, history of myocardial infarction, and pregnancy and lactation. Only 6 patients had classic migraine and were excluded in order to reduce bias. The patients were divided randomly into two equal groups. The mean headache severity in the patients before treatment as well as half an hour, one hour, and two hours after treatment was measured using the Verbal Numerical Rating Scale (VNRS) ranging from zero to ten.

After randomization of the patients into two groups, the first group was injected with 6 mg of Sumatriptan subcutaneously plus 200 cc of normal saline in 20 minutes and the second group received 400 mg of sodium valproate (Depakine, SANOFI AVENTIS, France) in 200 cc of normal saline (in 20 minutes) plus 2 cc of subcutaneous normal saline. Because intravenous sodium valproate in Iran is only available in 400-mg ampoules and previous studies have proved that 300-500 mg of intravenous sodium valproate is sufficient for subsiding pain,^18^^-^^20^ we chose 400 mg of sodium valproate. Before and 2 hours after treatment, the patients’ vital signs were check precisely. Headache severity was measured on admission and thereafter half an hour, one hour, and two hours after treatment based on the VNRS in the two groups separately. Other symptoms, including photophobia, phonophobia, nausea, and vomiting,  were assayed on admission and then 2 hours after treatment. The side effects of the drugs, including nausea, vomiting, facial paresthesia, and hypotension, were checked 2 hours after injection. An informed consent was obtained from all the patients, and the Ethics Committee of Yazd University of Medical Sciences approved the study.**

At the end of the trial, the gathered data were analyzed using SPSS 11.5 software and statistical tests (Chi-square, Mann-Whitney, Fisher, and Repeated Measure ANOVA tests). A P<0.05 was considered statistically significant. **

## Results

The sodium valproate group comprised 11 men and 34 women at a mean±SD age of 31.3±3.5 years, and the Sumatriptan group consisted of 12 men and 33 women patients at a mean±SD age of 30.1±3.1 years. The groups had no significant difference based on sex (P=0.809).**

**Figure 2:**
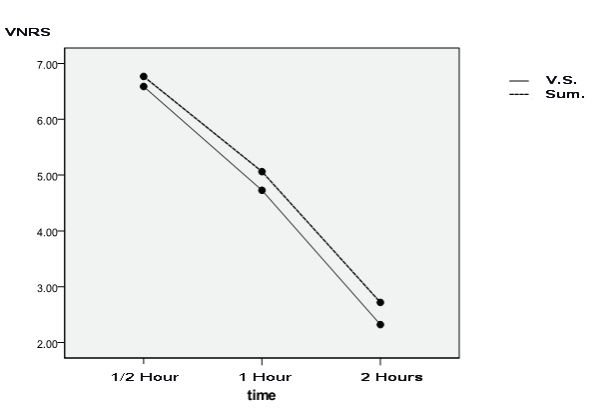
Comparison the effect of the drugs on reducing headache severity at similar time points according to the Repeated Measure ANOVA test. V.S.: Valproate sodium; Sum.: Sumatriptan; VNRS: Verbal Numerical Rating Scale

Table 1 shows the mean of headache severity before treatment as well as half an hour, one hour, and two hours after treatment in the sodium valproate and Sumatriptan groups, separately. Figure 2 demonstrates a comparison between the two drugs at the mentioned time points using the Repeated Measure ANOVA test.

**Table 1:** Comparison of the effect of the drugs on reducing headache severity at similar time points

**Time**	**Valproate** **mean±SD** ** (Median)**	**Sumatriptan** **mean±SD** ** (Median)**	**P value**
Before injection	8.16±1.62 (8)	9.11±0.91(9)	0.315
½ Hour	6.07±1.72 (6)	7.27±1.01 (7)	0.454
1 Hour	4.29±1.68 (5)	5.62±1.23 (6)	0.274
2 Hours	1.89±1.40 (2)	3.22±1.55 (3)	0.345

SD: standard deviation; Headache severity is shown by Mean VNRS.

In both groups, pain decrement at the mentioned time points compared to before injection was significant (P<0.001). Comparing these decrement rates in both groups at similar time points showed no significant difference, indicating the similar effect of sodium valproate and Sumatriptan on pain severity decrement. 

Table 2 depicts the rate of improvement in migraine-associated symptoms in the two groups and a comparison of these improvement rates between the two groups. According to this table, photophobia, phonophobia, nausea, and vomiting were improved significantly in the sodium valproate group, while only photophobia and vomiting were decreased significantly in the Sumatriptan group, denoting the advantage of sodium valproate in improving associated symptoms.

**Table 2:** Comparison of the effect of the drugs on associated symptoms

** Groups**** **Symptoms**	**Valproate**	**Sumatriptan**	**P value**
	**Number of patients (Improved)**	**Number of patients (Improved)**	
Photophobia	20 (14)	21 (9)	0.118
Phonophobia	17 (10)	16 (5)	0.166
Nausea	28 (12)	23 (5)	0.143
Vomiting	9 (7)	10 (8)	1

Table 3 illustrates the incidence rate of the side effects of the drugs in each group and a comparison of these rates between the two groups. The side effects of the drugs had been evaluated in the patients without the mentioned symptoms before drug administration. For example, in the sodium valproate group, 28 patients had nausea initially and were, therefore, excluded before drug administration and the remaining 17 patients were followed up  for nausea; 5 of these 17 patients had nausea after drug administration. No patient in the two groups initially had facial paresthesia or hypotension and other symptoms such as vertigo and blurred vision. Table 3 shows that nausea, vomiting, facial paresthesia, and hypotension were more significantly frequent in the Sumatriptan group than in the sodium valproate group. **

**Table 3:** Comparison of the side effects of the drugs between the two groups

** Groups**** **Symptoms**	**Valproate**	**Sumatriptan**	**P value**
Nausea/Vomiting	5/8*	12/12	0.049
Face paresthesia	1/45	9/45	0.015
Hypotension	0/45	8/45	0.006
Others	2/45	2/45	1
No side effect	37/45	14/45	<0.001

5/8* means that 8 patients in the sodium valproate group had no nausea and vomiting on admission (before drug administration). After drug injection, 5 patients out of 8 complained from nausea and vomiting.

## Discussion

One of the most acceptable drugs for the treatment of acute migraine attacks is Sumatriptan. Sumatriptan is a selective agonist of 5-hydroxy-tryptamine 1B and 1D (5-HT1B/1D) receptors and acts by constricting the meningeal blood vessels that are dilated, blocking the vasoactive neuropeptides that are released from the perivascular trigeminal sensory neurons, and reducing pain signal transmission in the trigeminal dorsal horn.^24^^,^^25^ The drug forms of  Sumatriptan are subcutaneous injection (4-6 mg), oral tablets (25 mg, 50 mg, and 100 mg) and nasal spray (5 and 20 mg).^26^ Based on previous studies, the rates of headache relief after the injection of 6 mg of subcutaneous Sumatriptan at one, two, and 24 hours are 71%, 79%, and 31%, respectively.^27^ The rates of relief from headache-associated symptoms (nausea, photophobia, and phonophobia) two hours after Sumatriptan injection are 76%, 71%, and 72%, respectively, and the rate of adverse effects within 24 hours after the injection of Sumatriptan (6 mg) is 44%.^27^ Sumatriptan, apart from its efficacy in treating acute migraine attacks, has the following serious contraindications: ischemic heart disease, prince metal angina, cerebrovascular disease, peripheral vascular disease, uncontrolled hypertension,^28^^,^^29^ familial hemiplegic migraine, pregnancy, and interaction with monoamine oxidase inhibitors and Ergotamine.^30^ The side effects of Sumatriptan include nausea, vomiting, fullness and rigidity in the neck, chest discomfort, odynophagia, otalgia, face and limb numbness, and paresthesia.^30^

Accordingly, given the side effects and prescription limitations of Sumatriptan, investigators have used other drugs for the acute treatment of migraine attacks. One of these alternatives is intravenous sodium valproate,^19^^,^^20^^-^^23^ which is an antiepileptic drug that acts by increasing the inhibitory effect of gamma-aminobutyric acid GABA on the hypothalamus neurons.^31^**

Previous studies have shown the prophylactic effect of sodium valproate on migraine attack prevention,^18^ and recent investigations into the effects of sodium valproate on relieving acute migraine attacks have yielded interesting results.^19^^,^^20^^-^^23^ Be that as it may, to our knowledge, there is no study in the existing literature to compare subcutaneous Sumatriptan and intravenous sodium valproate.**

The important results from the previous studies are: **

1. Sodium valproate has considerable effectiveness in the first hour (25-75%); these results are comparable with those of other drugs used in acute migraine attacks.
2. Sodium valproate dosages are variable; however, it seems that lower dosages (300-500 mg) also may lead to an appropriate response.
3. There are no significant side effects after sodium valproate administration.
4. There is a significant improvement in the symptoms associated with acute migraine attacks.
5. There is more improvement through intravenous sodium valproate for patients not receiving prophylactic sodium valproate compared to patients on sodium valproate prophylaxis.^31^

The salient findings in our current study are: **

1. Comparison of the mean headache severity decrement between sodium valproate and Sumatriptan at half an hour, one hour, and 2 hours after administration showed that sodium valproate was as effective as Sumatriptan for headache relief.
2. Sodium valproate was more effective than Sumatriptan in decreasing the associated symptoms.
3. The side effects of sodium valproate were significantly fewer than those of Sumatriptan. 
In previous studies that similarly used intravenous sodium valproate, no significant side effects were reported.^19^^-^^22^ The only report for side effects was made by the Shahien R et al.^23^ study (2011): photophobia (67%); unilateral pain (50%); vomiting (41%); phonophobia (39%); and pulsatile pain (36%). In that study, the loading dose of sodium valproate was 900-1200 mg. Consequently, the difference between the dosage in that study and ours (400 mg) may explain the conflicting results.
4. The prescribed dose of sodium valproate, i.e., 400 mg, seems appropriate for relieving acute migraine attacks.
5. In the Sumatriptan group, the improvement rates of nausea, photophobia, and phonophobia were very low compared to those reported previously.^27^ It seams that the main reasons for this discrepancy are genetic and ethnic differences.
6. All the patients without nausea and vomiting who received Sumatriptan developed nausea and vomiting. Thus, it seems that sodium valproate may be more effective than Sumatriptan in patients presenting without nausea and vomiting. 

Given that Sumatriptan has more side effects and administration limitations, the following findings of our study highlight the advantage of sodium valproate over Sumatriptan in the treatment of acute migraine attacks:

1. Sodium valproate has similar effectiveness compared to Sumatriptan.
2. Sodium valproate is more efficacious in alleviating headache-associated symptoms.
3. Sodium valproate can replace Sumatriptan in patients with contraindications for Sumatriptan use.
4. Sodium valproate has fewer side effects.

## Conclusion

Our study suggests that 400 mg of intravenous sodium valproate is effective in the treatment of acute migraine headache, particularly in patients not on sodium valproate prophylaxis or in patients with contraindications for Sumatriptan use. **
